# A Rare Cause of Post-traumatic Right Lower Extremity Swelling in an Adolescent Male

**DOI:** 10.7759/cureus.17726

**Published:** 2021-09-05

**Authors:** Adebayo Adeyinka, Yutika Mandal, Hasina Mohammad Ashraf, Louisdon Pierre, Noah Kondamudi

**Affiliations:** 1 Pediatrics, The Brooklyn Hospital Center, Brooklyn, USA; 2 Pediatric Critical Care Medicine, The Brooklyn Hospital Center, Brooklyn , USA; 3 Pediatrics, The Brooklyn Hospital, New York, USA

**Keywords:** arterio-venous fistula, lower limb trauma, adolesecent, limb swelling, ct angiogram

## Abstract

Unilateral extremity swelling after trauma usually results from acute musculoskeletal or orthopedic injuries. Worsening of swelling raises concern for compartment syndrome or vascular injury. Time-sensitive diagnosis and interventions are needed to avoid life- or limb-threatening consequences. In this report, we highlight the case of a 16-year-old male who presented with unilateral lower extremity pain and swelling, one week after a motor vehicle accident. Thorough evaluation and appropriate imaging detected the presence of an abnormal communication between the muscular branch of the anterior tibial artery and the vein. Arteriovenous fistulas (AVFs) are usually acquired and caused by penetrating trauma or iatrogenic procedures. They are rarely associated with blunt trauma. It is important to determine the degree of flow within the communication, as high flow lesions are associated with severe complications such as limb ischemia and heart failure. This report highlights the evaluation and management of a patient with delayed post-traumatic unilateral extremity swelling that eventually resulted in the diagnosis of a low-flow AVF amenable to conservative management, resulting in complete resolution of his symptoms.

## Introduction

Unilateral extremity swelling after trauma is usually a result of acute musculoskeletal and/or orthopedic injuries. Worsening or persistent swelling may be due to a variety of causes. Compartment syndrome and vascular injuries are of particular concern, as delays in recognition can lead to life or limb-threatening complications. Arteriovenous fistulas (AVFs) are an uncommon cause of unilateral limb swelling and are usually caused by penetrating trauma, post-surgical or vascular interventions, and inflammatory response [1]. Only 10% of cases of AVFs are attributed to blunt trauma [2]. In nearly 50% of AVF cases, the clinical symptoms are very non-specific, contributing to the challenge in making this diagnosis [3]. Patients with AVFs may have a variety of clinical manifestations from being completely asymptomatic and detected as an incidental finding to life-threatening cardiac failure among patients with high flow malformations [4]. Although rare, the diagnosis of AVF should always be considered in a high-impact extremity trauma [5]. We report the case of an adolescent male who presented with unilateral right lower extremity swelling. We described in detail his diagnostic workup, hospital course, and management until resolution of his symptoms.

## Case presentation

A 16-year-old male presents to the emergency department (ED) with complaints of pain, swelling, and difficulty bearing weight on the right lower extremity. A week prior to this presentation, the patient’s bike collided with a car resulting in an impact injury to the lateral aspect of his right knee. X-ray of the right ankle done at the local urgent care center was normal and he was discharged home with a diagnosis of a right ankle sprain. The pain and swelling persisted and progressively got worse over the next week, prompting the visit to our ED.

**Figure 1:**
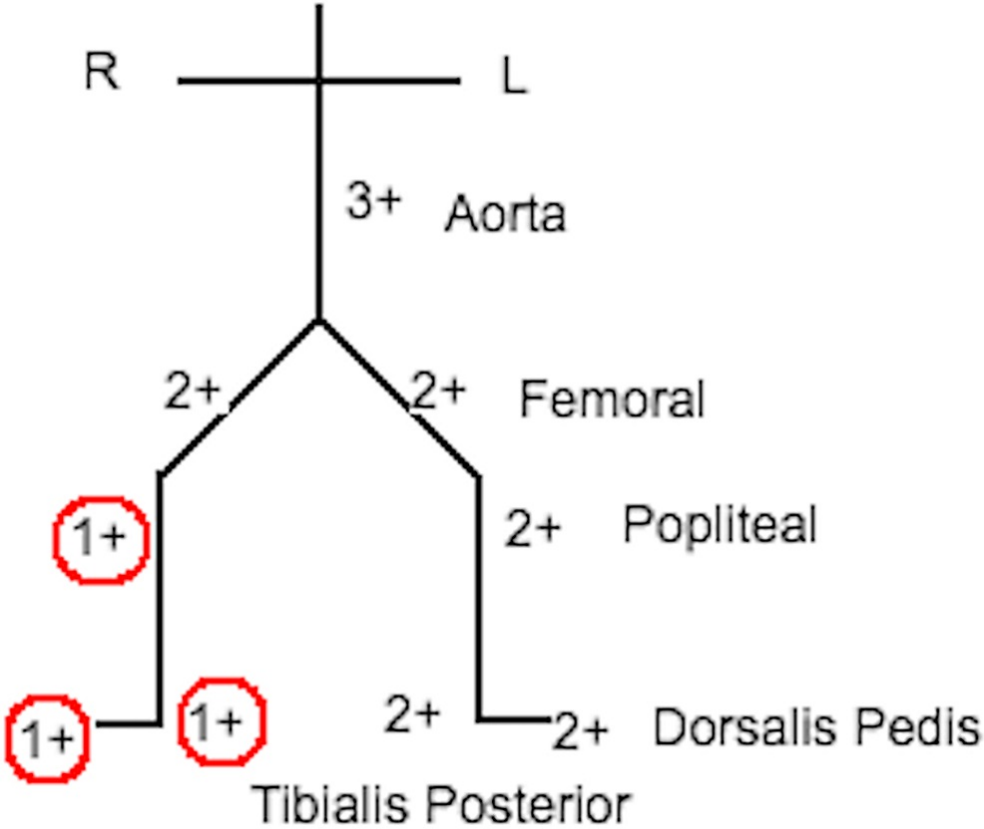
Diagrammatic representation of lower limb pulsations

In our ED, the patient appeared to be in moderate discomfort, unable to bear weight and has an ankle brace wrapped around his right ankle. Vital signs on presentation: temperature = 99.3 F, heart rate = 113 beats per minutes (tachycardia), blood pressure = 117/73 mmHg , respiratory rate = 18 breaths per minute, pulse oximetry was 98% on room air. On physical examination, there was moderate swelling of the right foot and ankle area with significant edema extending to the lower third of the leg, along with severe tenderness that is exaggerated upon moving the toes. Evaluation of pulses in both lower extremities revealed a noticeable reduction in the right popliteal, posterior tibialis, and dorsalis pedis arteries (Figure 1). The right foot felt colder and paler compared to the left foot without any erythema or bluish discoloration of the toes.

The surgery team was consulted due to the suspicion for possible compartment syndrome and vascular compromise and the patient was admitted to the in-patient unit for further workup and management. Doppler sonogram of the right lower extremity showed no evidence of deep vein thrombosis or vascular compromise.

**Figure 2:**
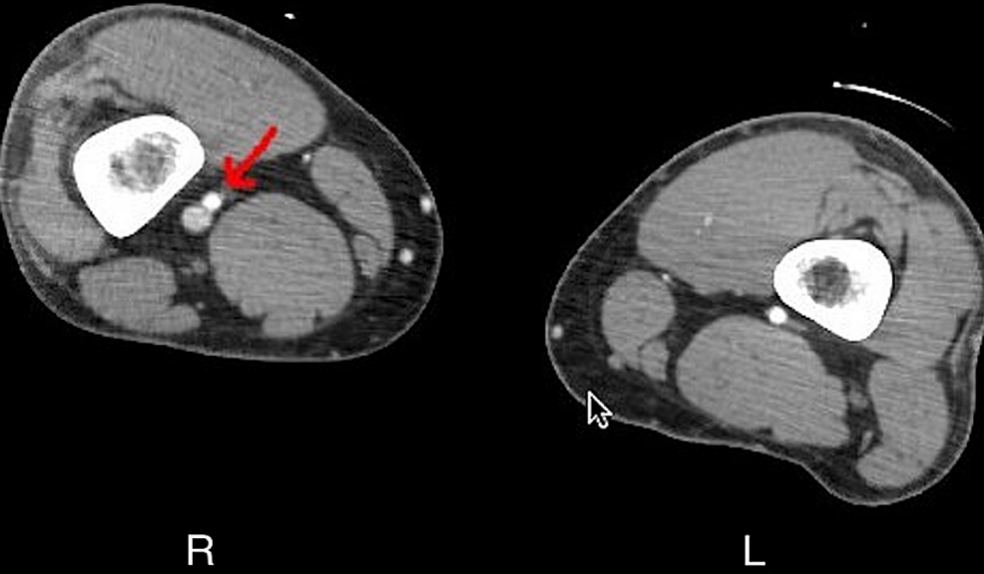
CT angiography with contrast of the lower extremities showing contrast in the vein adjacent to the artery in the right extremity (red arrow)

**Figure 3:**
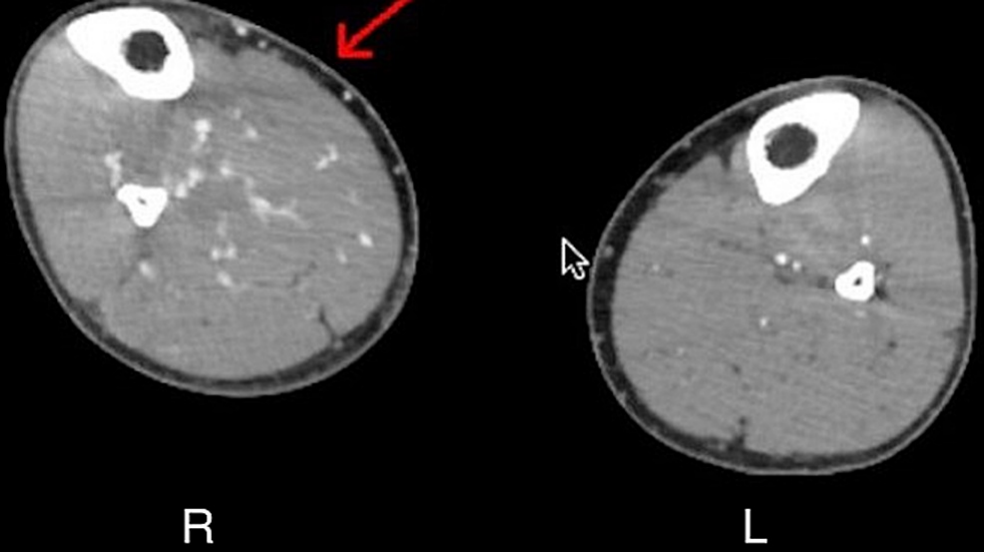
CT angiography with contrast of the lower extremities showing multiple contrast-enhanced receiving veins in the right extremity compared to the left extremity at the same level

The following day the patient started complaining of complete loss of sensation in the extremity from around the knee to the toes. CT angiography of the right lower extremity with contrast revealed contrast in the adjoining vein adjacent to the artery along the area of trauma on the right side compared to the healthy left lower extremity (Figure 2). The next diagram shows multiple contrasts enhance receiving veins in the right lower extremity as compared to the left lower extremity (Figure 3).

The interventional radiologist on a careful review of the CT angiogram raised the suspicion of a possible traumatic AV fistula and recommended selective IR angiography of the right lower extremity. This study is diagnostic and at the same time allows for therapeutic intervention if needed.

This study clearly defined the abnormal communication between the muscular branch of the right anterior tibial artery and the vein (Video 1). No immediate vascular intervention was required at this time as this was a low flow of fistulous communication. The patient reported improvement of his symptoms following the procedure, manifesting less pain and return of skin sensation. He received physical therapy during his hospital stay, which also attributed to his recovery of weight-bearing and range of motion in the affected right lower extremity. He was subsequently discharged home with instructions to continue physical therapy and follow up with interventional radiology.

**Video 1:** Selective IR angiography of the right lower extremity showing a retrograde flow of contrast from the muscular branch of the anterior tibial artery to the anterior tibial vein.

## Discussion

Unilateral limb pain in children has a broad differential and accurate diagnosis is important to prevent life and limb-threatening complications. Some of the conditions that present with unilateral limb pain and swelling are listed in Table 1 [6].

The clinical presentation of AVF is variable depending on the location. They may have extremity swelling, ischemic pain, bleeding, or can present with pulsatile swelling with a palpable thrill and audible bruit on auscultation at the site of the AVF [7]. The physical exam finding of poor pulses in the right lower extremity drove further work up to evaluate for a vascular injury eventually resulting in the diagnosis of an AVF affecting the right tibial vessels.

Arteriovenous malformations and fistulas are aberrant connections between arteries and veins. Most AV malformations are congenital and most AVFs are acquired [8,9]. In AVFs, blood from the high-pressure arterial circuit gets shunted to low-pressure venous circulation without a capillary bed in between. The most frequently encountered AVFs are the ones created to facilitate vascular access for children with end-stage renal disease that undergo regular hemodialysis. These may also occur during attempts (iatrogenic) at vascular access when communication is established inadvertently between the artery and adjoining vein [10]. Penetrating trauma accounts for 90% of traumatic fistulae and is mostly from gunshot wounds [11]. The proposed pathogenesis of trauma-induced AVFs is that the initial formation of hematoma leads to adhesions between the adjacent vessels and results in an anomalous connection between the artery and the vein [12]. This phenomenon is rarer in pediatric patients with non-penetrating trauma [12,13]. Only 10% of all AVFs were due to blunt trauma [5].

**Table 1:** Differential diagnosis for unilateral limb pain and swelling

System	Pathology
Musculoskeletal	Osteomyelitis, Septic arthritis, Fractures, Soft tissue injury, Hemarthrosis, Leukemia, Malignancy
Vascular	Vaso-occlusive crisis - sickle cell disease, Acute thrombophlebitis, Acute deep vein thrombosis, Vascular injuries
Dermatologic	Local allergic reactions, Cellulitis

AVF as a diagnostic consideration would be quite low in our patient considering his age (adolescent), mechanism of injury (blunt), and the duration between injury and symptomatic presentation - one week. AVFs have the potential to be missed during the initial presentation due to the focus on managing the primary injuries [5]. The presenting symptoms of AVF depend on the location and the degree of shunting in the lesion. Our patient presented with symptoms of pain and swelling of his right leg and was found to have tachycardia and reduced peripheral pulses. It was difficult to determine the origin of the extremity pain, as it could be attributed either to the primary injury, fracture, compartment syndrome, or AVF formation. At the time of presentation, traumatic AVF was very low on the differential diagnosis. The extremity swelling could be attributed to the ankle brace that the patient came with but may also be the result of the AVF. There was also a significant concern for acute compartment syndrome. Compartment syndrome is a limb-threatening emergency that needs to be recognized and treated emergently. The classic signs of the 5Ps (pain, pallor, pulselessness, paresthesias, and poikilothermia) described in compartment syndrome are thought to be unreliable with poor sensitivity and specificity [14].

The timing between the trauma event and the occurrence of symptoms can be variable and can range from a few days to several months or years [11]. Our patient presented one week after the injury. AVF should always remain in consideration when a patient presents with localized symptoms. 

Imaging studies are the best modality to detect vascular anomalies related to traumatic injuries. Duplex ultrasound can demonstrate the AVF if it is in a relatively superficial location [15]. The initial choice of imaging is duplex ultrasound due to its non-invasive nature and low cost [16]. However, it is operator-dependent and may not be available in all settings. MRI can provide more detailed information about the anomaly, relationship to adjacent structures, and flow characteristics [17,18]. Angiography is a more advanced study and helps in the classification of vascular lesions. Both CT and Magnetic resonance angiography can demonstrate AVFs but the gold standard is selective angiography of the affected vessel, which was performed on our patient [19].

The choice of treatment depends on the size of the AVF, the extent of blood-flow shunting, and signs of progression. High-flow AVFs are at increased risk of leading to heart failure and limb ischemia due to the diversion of the blood flow. A conservative approach with observation and close follow-up is acceptable in the absence of indications for surgery. Indications for surgery include hemodynamic instability, cardiac decompensation, worsening ischemia, or failure to regress spontaneously within two weeks [20]. The goal of surgical interventions is to close the AVF while maintaining essential blood flow to the tissues. Options for closure include open surgery or IR-guided endovascular approach with covered stent placement or embolization [3]. Our patient was managed conservatively as the AVF was a low flow lesion and he showed steady improvement during the hospital course. The role of possible feeding artery spasm during microcatheter insertion toward clinical improvement is unknown.

## Conclusions

AVFs sustained from blunt trauma are rare among children. For patients presenting with unilateral extremity pain and swelling after trauma, AVF should always be considered a part of the differential diagnosis, which is quite broad. Imaging studies, such as duplex ultrasound, CT, and MR angiography, can diagnose an AVF, but selective IR angiography is the gold standard. Management ranges from a conservative approach to IR embolization or surgical ligation, depending on the degree of flow across the fistula. Early recognition and quick intervention are necessary to avoid the potential of developing severe complications like limb ischemia and high output cardiac failure.

## References

[1] Holt GR, Holt JE, Cortez EA, Thornton WR, Young WC (1980). Traumatic facial arteriovenous malformations. Laryngoscope.

[2] Mulatti GC, Queiroz AB, daSilva ES (2013). Arteriovenous fistulas - diagnosis and management. Traumatic Arteriovenous Fistula.

[3] Nagpal K, Ahmed K, Cuschieri R (2008). Diagnosis and management of acute traumatic arteriovenous fistula. Int J Angiol.

[4] Hyodoh H, Hori M, Akiba H, Tamakawa M, Hyodoh K, Hareyama M (2005). Peripheral vascular malformations: imaging, treatment approaches, and therapeutic issues. Radiographics.

[5] Roth P, Heiss C, Koshty A, Niemann B, Boening A (2014). Posttraumatic arteriovenous fistula of the distal posterior tibial artery as cause of delayed wound healing in an unrecognized arterial injury. Thorac Cardiovasc Surg Rep.

[6] Tse SM, Laxer RM (2006). Approach to acute limb pain in childhood. Pediatr Rev.

[7] Sarac M, Marjanović I, Jevtić M, Misović S, Zoranović U, Rusović S (2011). Endovascular repair of posttraumatic multiple femoral-femoral and popliteal-popliteal arteriovenous fistula with Viabahn and excluder stent graft. Vojnosanit Pregl.

[8] Uller W, Alomari AI, Richter GT (2014). Arteriovenous malformations. Semin Pediatr Surg.

[9] Madani H, Farrant J, Chhaya N, Anwar I, Marmery H, Platts A, Holloway B (2015). Peripheral limb vascular malformations: an update of appropriate imaging and treatment options of a challenging condition. Br J Radiol.

[10] Muller DW, Shamir KJ, Ellis SG, Topol EJ (1992). Peripheral vascular complications after conventional and complex percutaneous coronary interventional procedures. Am J Cardiol.

[11] Fox CJ, Gillespie DL, O'Donnell SD (2005). Contemporary management of wartime vascular trauma. J Vasc Surg.

[12] Kotagal M, Reiss A, Vo N, Feldman K, Drugas G, Avansino JR (2012). Iatrogenic arteriovenous fistula in the arm in an infant: diagnostic and therapeutic considerations. J Clin Ultrasound.

[13] Ramdass MJ (2019). Extreme traumatic arteriovenous fistula of the upper limb. Clin Case Rep.

[14] Ulmer T (2002). The clinical diagnosis of compartment syndrome of the lower leg: are clinical findings predictive of the disorder?. J Orthop Trauma.

[15] Davison BD, Polak JF (2004). Arterial injuries: a sonographic approach. Radiol Clin North Am.

[16] McCafferty I (2015). Management of low-flow vascular malformations: clinical presentation, classification, patient selection, imaging and treatment. Cardiovasc Intervent Radiol.

[17] Samadi K, Salazar GM (2019). Role of imaging in the diagnosis of vascular malformations vascular malformations. Cardiovasc Diagn Ther.

[18] Dubois J, Alison M (2010). Vascular anomalies: what a radiologist needs to know. Pediatr Radiol.

[19] Ares WJ, Jankowitz BT, Tonetti DA, Gross BA, Grandhi R (2019). A comparison of digital subtraction angiography and computed tomography angiography for the diagnosis of penetrating cerebrovascular injury. Neurosurg Focus.

[20] Jayroe H, Foley K (2013). Retrospective Analysis of 271 Arteriovenous Fistulas as Vascular Access for Hemodialysis. Indian J Nephrol.

